# Effectiveness of the MOVE! Multidisciplinary Weight Loss Program for Veterans in Los Angeles

**DOI:** 10.5888/pcd10.120325

**Published:** 2013-07-03

**Authors:** Maria Romanova, Li-Jung Liang, Max L. Deng, Zhaoping Li, David Heber

**Affiliations:** Author Affiliations: Maria Romanova, Max L. Deng, VA Greater Los Angeles Healthcare System, Los Angeles, California; Li-Jung Liang, David Heber, David Geffen School of Medicine at University of California, Los Angeles, California. Dr Li is also affiliated with the Center for Human Nutrition, David Geffen School of Medicine at University of California, Los Angeles.

## Abstract

**Introduction:**

The purpose of this study was to evaluate the effectiveness of the MOVE! Weight Management Program for Veterans (MOVE!) in achieving weight loss in veterans who attended the multidisciplinary weight management program in the VA Greater Los Angeles Healthcare System.

**Methods:**

From April 1, 2006, to December 31, 2009, 382 veterans enrolled in the MOVE! program; 377 veterans attended at least 3 group sessions and were included in this study. All veterans were encouraged to complete 8 weekly group sessions on nutrition, lifestyle changes, and behavior modification in a group setting led by a multidisciplinary team. After completing the session, veterans had the option of continuing with a support group that meets monthly. The change in weight from 1 year pre-enrollment in MOVE! to 1, 2, and 3 years postenrollment was analyzed.

**Results:**

Veterans gained 1.4 kg per year (standard error [SE] = 0.47, *P* = .003) before enrolling in MOVE!. One year after the enrollment participants lost on average 2.2 kg (SE = 0.42; *P *< .001). The pre-enrollment slope for weight change was significantly different from the postenrollment slope.

**Conclusion:**

Findings from this study support the need for a long-term weight management program such as MOVE! in primary care settings to assist overweight and obese VA patients in achieving and maintaining weight loss to reduce the risk and progression of age-related chronic diseases such as diabetes and heart disease.

## Introduction

Obesity is associated with higher rates of hypertension, dyslipidemia, diabetes mellitus, degenerative joint disease, and coronary artery disease (1). These diseases and their treatment may increase the risk of weight gain and make weight loss more challenging (2–4). Obesity and overweight are also associated with poor quality of life, premature death, and increased health care costs. Lifestyle factors including excess intake of calories and low levels of physical activity are central causes of obesity, and the combination of increased physical activity, reduced caloric intake, and other behavior modifications results in weight loss for overweight people (5).

The prevalence of overweight and obesity in veterans is higher than in the general population but may be similar when the demographics of the veteran population are considered (6–9). Programs that can help participants achieve weight losses as small as 5% of their body weight can help them reduce the risk of chronic conditions such as type 2 diabetes (10). Intensive interventions such as the Diabetes Prevention Program and the Look Ahead trials have been effective in helping participants achieve clinically significant weight loss (11,12).

Adapting these research programs into ambulatory programs that can be feasibly delivered through the US Department of Veterans Affairs (VA) is challenging; the Veterans Health Administration (VHA) provides care to almost 6 million veterans through a nationwide network (13). In response to the challenge of obesity in VHA, the MOVE! Weight Management Program for Veterans (MOVE!) program was piloted between 2002 and 2004 and was nationally implemented in 2006. By 2009, nearly all (98.7%) of the 155 medical centers in VHA reported having MOVE! programs in place (14). In the Greater Los Angeles VA, a multidisciplinary MOVE! weight management program has been in place since April 2006. However, a recent survey of VA patients in 4 Western states (15) indicated that less than 5% of veterans who were candidates for MOVE! participated. Therefore, optimizing this program and increasing general awareness of its beneficial long-term results could be used to market this no-cost program to more veterans. In that cross-sectional and longitudinal study (15), women were more likely to participate than men but were less likely to have clinically relevant weight loss. Therefore, improvements to the MOVE! program can be made in different locations, and different features can be emphasized. Evaluation of components that appear to be working among the various sites of this VA program is needed.

The purpose of this study was to determine significant predictors of weight loss at 3 years in veterans who attended the MOVE! multidisciplinary weight management program at the VA Greater Los Angeles Healthcare System (VAGLAHS). Findings from this study can provide evidence to support the need for a lifestyle modification program such as MOVE! in primary care settings to assist overweight and obese veterans in managing their weight.

## Methods

### Participants

This study was approved by the VAGLAHS institutional review board. All charts of veterans enrolled in MOVE! from April 1, 2006, to December 31, 2009, were reviewed. Veterans were referred to MOVE! by their primary care provider if they were obese (body mass index [BMI] >30 kg/m^2^) or overweight (BMI 25.0−29.9 kg/m^2^) and had comorbid conditions such as hypertension, hyperlipidemia, and type 2 diabetes.

### MOVE! program

The VA National Center for Health Promotion and Disease Prevention (NCP) developed MOVE! to provide a standardized format for weight management (16). To disseminate the program, NCP created handouts for veterans, training modules for staff, curricula for group sessions, weight management assessment tools, and methods for electronic tracking of participation in program activities. Each facility was permitted to determine its own methods to identify veterans for the program and the types and extent of offerings in the program. All veterans enrolled into the MOVE! program at VAGLAHS attended a 2-hour nutrition class led by a dietitian. Veterans then had the option of participating in 8 weekly multidisciplinary education sessions in a group setting. Those sessions lasted 1 hour and were led by physicians, dietitians, physical and recreational therapists, and psychologists. Group sessions focused on a particular theme and included nutrition, physical activity, and behavioral health perspectives.

Typically, during the first encounter, staff provided an overview of the program and instructed veterans to complete a 23-item questionnaire on their diet, physical activity, health status, and prior weight loss attempts. An individualized report was then generated; it included a list of recommended print-ready materials on nutrition, physical activity, and healthy behavior changes available from the MOVE! website (www.move.va.gov). Veterans were encouraged to set realistic and attainable goals and were instructed that a sustainable rate of weight loss was about 0.5 to 1 kg per week. In addition to group-based sessions, a one-on-one counseling session was provided as needed.

### Weight measurement

Pre-enrollment and postenrollment weight was obtained from veterans’ electronic medical records. Weight was measured and entered by the medical staff when the veteran attended his or her regularly scheduled medical appointment. Pre-enrollment weights consisted of measurements from 1 year before enrollment in the program, and postenrollment weight consisted of measurements available in the veteran’s medical record postenrollment in MOVE!. A 3-month window was used for pre-enrollment weights and at 1-, 2-, and 3-year follow-up, whereas a 1-month window was used at 3- and 6-month follow-up to increase the likelihood of obtaining a valid weight value from the medical record. Demographic characteristics, baseline weight, and comorbid conditions for the MOVE! participants were obtained, and all active medical diagnoses in patients’ medical records were included in the analysis.

### Statistical analysis

Linear mixed-effects regression models with a piecewise linear function specified for each study participant (ie, participant-level random effects) were used to evaluate changes in body weight before and after enrollment in MOVE!. Covariates were age, sex, and linear piecewise-time segments (4 slopes: before MOVE!, and 0 to 1 year, >1 to 2 years, and >2 years post-MOVE!). The advantage of using a piecewise approach was that we could estimate the slopes simultaneously because we expected the time trends to be different for different time windows. The slopes before MOVE! versus 1 year post-MOVE! were estimated and compared through model contrasts. The model included participant-level random effects (ie, individual’s intercept and 4 slopes) to account for correlation among repeated observations among participants. For exploratory purposes, we conducted several subgroup analyses for the selected comorbid conditions using the same modeling approach as in the main analysis with the following additional terms in the model: condition (yes vs no) and condition-by-time interaction terms (1 for each time segment). All statistical analyses were conducted using SAS version 9.2 for Windows (SAS Institute, Inc, Cary, North Carolina).

## Results

### Demographic characteristics

A total of 382 veterans enrolled in the MOVE! program (Table 1). Approximately 88% of the veterans were men; the average age for the veterans at time of enrollment was 60 (standard deviation [SD] = 11.1; range, 25–92), and 6.5% of them were older than 75. The average body weight at time of enrollment was 110 kg (SD = 23.6 kg; range, 54.7–194.2 kg), and more than 80% of participants had a BMI of 30 kg/m^2^ or higher. More than 70% of veterans had hyperlipidemia or hypertension; approximately 40% had mood disorder or type 2 diabetes; 26% to 33% had a substance abuse condition, osteoarthritis, obstructive sleep apnea, or posttraumatic stress disorder; and less than 20% had gastroesophageal reflux disease, anxiety, coronary artery disease, or schizophrenia.

**Table 1:** Demographic Characteristics and Comorbid Conditions of Veterans (N = 382),^a^ MOVE! Weight Management Program for Veterans, Los Angeles, California, 2006–2009

Characteristic	Value
**Age**	
Mean, y (SD)	60 (11.1)
Median, y (range)	62 (25–92)
No. missing (%)	17 (4.5)
**Sex, n (%)**	
Female	30 (7.9)
Male	335 (87.7)
Unknown/missing	17 (4.5)
**Weight status at enrollment (BMI, kg/m^2^), n (%)**	
Normal weight (<25)	10 (2.6)
Overweight (≥25 and ≤29.9)	63 (16.5)
Obese (≥30 and ≤39.9)	233 (61.0)
Morbid obesity (≥40.0)	76 (19.9)
**Weight at enrollment, kg**	
Mean (SD)	110.1 (23.6)
Median	106.4
Range	54.7–194.2
**Race, n %**	
White	53 (13.9)
Black	48 (12.6)
Mexican American	2 (0.5)
Unknown/missing	279 (73.0)
**Comorbid conditions, n (%)**	
Hyperlipidemia	276 (72.3)
Hypertension	270 (70.7)
Mood disorder	163 (42.7)
Type 2 diabetes	152 (39.8)
Substance abuse	126 (33.0)
Osteoarthritis	103 (27.0)
Obstructive sleep apnea	102 (26.7)
Posttraumatic stress disorder	100 (26.2)
Gastroesophageal reflux disease	75 (19.6)
Anxiety	74 (19.4)
Coronary artery disease	47 (12.3)
Schizophrenia	36 (9.4)

a Study participants may have multiple comorbid conditions.

### Weight change pre-MOVE! and post-MOVE! enrollment

MOVE! participants who attended at least 3 sessions were included in the weight-change analysis (N = 377). Results from the mixed-effects piecewise regression model, adjusting for age and sex, indicated that veterans gained an average of 1.4 kg per year (standard error [SE] = 0.47, *P* = .003) before they enrolled in MOVE! (Table 2). After enrolling in the program, veterans on average lost 2.2 kg per year (SE = 0.42; *P *< .001) 1 year post-MOVE!. The difference in slopes pre-MOVE! and 1 year post-MOVE! was significant (*P *< .001). We observed a nonsignificant increase in weight (0.95 kg; SE = 0.57) during the second year post-MOVE! and a nonsignificant decrease in weight (0.54 kg; SE = 0.76) after the second year of MOVE! (Figure). Older age (*P* = .01) and female sex (*P *< .001) were significantly associated with lower body weight at baseline.

**Table 2:** Results from Regression Model^a^ on Weight Change, MOVE! Weight Management Program for Veterans, Los Angeles, California, 2006–2009

Parameter	Mean Weight Change, kg (Standard Error)	*P* Value
**Age, y**	−0.27 (0.11)	.01
**Male sex**	18.3 (4.31)	<.001
**Slope by period**		
Pre-MOVE!	1.44 (0.47)	.003
**Post-MOVE!**		
0 to 1 year	−2.19 (0.42)	<.001
>1 to 2 years	0.95 (0.57)	.099
>2 years	−0.54 (0.76)	.48

a Analysis was adjusted for age at enrollment and sex.

**Figure:**
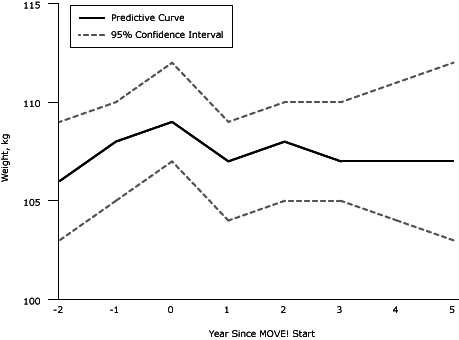
Weight change pre- and postenrollment in the MOVE! Weight Management Program for Veterans, Los Angeles, California, 2006–2009. **Table:** 

Year Since MOVE! Start	Weight, kg (95% Confidence Interval)
**−2**	106 (103–109)
**−1**	108 (105–110)
**0**	109 (107–112)
**1**	107 (104–109)
**2**	108 (105–110)
**3**	107 (105–110)
**4**	107 (104–111)
**5**	107 (103–112)

A significant weight loss (1.68 to 2.89 kg/y) was observed 1 year post-MOVE! among veterans who did not have comorbid conditions, except for those with hyperlipidemia or hypertension (Table 3). A significant weight reduction 1 year post-MOVE! was observed for veterans who had the following comorbid conditions: osteoarthritis (1.67 kg/y), type 2 diabetes (2.02 kg/y), obstructive sleep apnea (2.79 kg/y), gastroesophageal reflux disease (3.47 kg/y), hyperlipidemia (2.54 kg/y), and hypertension (2.57 kg/y). One hundred sixty-two (42%) veterans had at least 1 psychiatric condition (anxiety, posttraumatic stress disorder, or schizophrenia), and 287 (75%) veterans had 3 or more comorbid conditions. Veterans who did not have a psychiatric condition showed significant weight reduction 1 year post-MOVE! (2.89 kg/y, *P*< .001); the difference in 1-year post-MOVE! slopes for veterans without versus with psychiatric conditions was not significant (slopes: 1.25 vs 2.89 kg/y, *P* = .052). Similarly, veterans who had 3 or more comorbid conditions showed significant weight reduction 1 year post-MOVE! (2.05 kg/y; SE = 0.48; *P*< .001). No significant difference in 1-year post-MOVE! slopes between veterans who had 3 or more comorbid conditions versus those who had fewer than 3 comorbid conditions was found.

**Table 3:** Weight Loss Pre-Enrollment and Postenrollment, by Comorbid Condition,^a^ MOVE! Weight Management Program for Veterans, Los Angeles, California, 2006–2009

Comorbid Condition	Without Condition, kg/y, Slope (SE)		With Condition, kg/y, Slope (SE)	
	Pre-MOVE!	1 Year Post-MOVE!	Pre-MOVE!	1 Year Post-MOVE!
Osteoarthritis^b^	1.93 (0.58)^c^	−2.20 (0.50)^d^	0.24 (0.86)	−1.67 (0.79)^c^
Mood disorder^b^^,^ e	1.18 (0.66)	−2.80 (0.57)^d^	1.64 (0.70)^c^	−1.15 (0.63)
Coronary artery disease^b^	1.56 (0.52)^c^	−2.31 (0.46)^d^	0.37 (1.30)	−1.53 (1.17)
Type 2 diabetes^b^^,^ e	1.43 (0.63)^c^	−2.07 (0.55)^c^	1.36 (0.75)	−2.02 (0.66)^c^
Posttraumatic stress disorder^b^^,^ e	1.13 (0.57)^c^	−2.24 (0.50)^c^	2.07 (0.90)^c^	−1.53 (0.82)
Substance abuse	1.01 (0.59)	−2.42 (0.52)^d^	2.17 (0.83)^c^	−1.32 (0.73)
Obstructive sleep apnea^b^^,^ e	1.38 (0.57)^c^	−1.76 (0.50)^c^	1.44 (0.90)^c^	−2.79 (0.80)^c^
Gastroesophageal reflux disease^b^^,^ e	1.20 (0.55)^c^	−1.68 (0.48)^c^	2.11 (1.03)^c^	−3.47 (0.93)^c^
Hyperlipidemia^e^	1.91 (1.01)	−0.54 (0.85)	1.25 (0.55)^c^	−2.54 (0.49)^d^
Hypertension^e^	1.09 (0.99)	−0.54 (0.83)	1.49 (0.55)^c^	−2.57 (0.49)^d^
Psychiatric diagnoses combined^b^^,^ e	1.53 (0.64)^c^	−2.89 (0.55)^d^	1.33 (0.71)	−1.25 (0.64)
≥3 Comorbid conditions^b^^,^ e	1.05 (1.15)	−2.05 (0.93)^c^	1.47 (0.53)^c^	−2.05 (0.48)^d^

Abbreviation: SE, standard error.

a All models were adjusted for age and sex.

b Significant difference in pre-MOVE! and post-MOVE! slopes for veterans without comorbid condition was observed.

c *P *< .05; slope was significantly different (greater or lower) from zero.

d *P *< .001; slope was significantly different (greater or lower) from zero.

e Significant difference in pre-MOVE! and post-MOVE! slopes for veterans with comorbid condition was observed.

## Discussion

We assessed treatment effects of MOVE! in a large sample of overweight and obese veterans by comparing the pre-MOVE! and post-MOVE! weight trajectory of veterans. Results indicated that veterans gained an average of 1.4 kg per year before enrolling in MOVE! and lost an average of 2.2 kg per year after enrolling in MOVE!; enrollment in MOVE! appeared to prevent further weight gain. Our results are consistent with those reported by Dahn et al (17), who assessed the trajectory of change in weight postintervention (3, 6, and 12 months postenrollment) from a preintervention period (1, 3, and 5 years before enrollment). The sample consisted of 862 veterans participating in MOVE! at the Miami VA. All veterans participated in a 2-hour self-management support session, which involved completion of a self-assessment questionnaire and a nutrition education group session. After completing the self-management support session, veterans had the option of continuing with supportive group sessions, which included 10-weekly group sessions led by a multidisciplinary team.

Williamson and colleagues (18) identified behavioral and dietary components as key parts in adherence to a weight management program. Behavioral adherence, including attendance of counseling sessions and self-monitoring, predicted reductions in body weight, waist circumference, and body fat (19). In our VAGLAHS MOVE! program, we aimed to create an estimated 500-calorie deficit daily in a program tailored to individual needs based on medical history, weight and weight management history, motivational factors, barriers to modifying physical activity, diet and weight-related behavior, and veterans’ readiness to change these behaviors. Staff assisted the patient with setting 1 to 3 specific short-term nutrition, physical activity, or behavior-change goals and supported the veteran weekly for 8 weeks and then through attendance of monthly weigh-ins and lectures. In this study, participants who attended the MOVE! program session at least 3 times demonstrated significant weight loss in 1 year and 2 years, suggesting the need for ongoing support to achieve long-term weight management success in a veteran population.

Multimorbidity was found in all age groups in this study. Major consequences of multimorbidity are disability and functional decline, poor quality of life, and high health care costs (20,21). VHA users have more physical and mental health conditions than people in community health care settings (22,23). Obesity-associated conditions such as hypertension, diabetes, ischemic heart disease, and arthritis are also prevalent in the VHA population (8,22). Lifestyle interventions result in reduction of obesity comorbidities, including mobility improvement (24,25). Long-term weight loss in epidemiological studies was associated with reduced risk of type 2 diabetes and may be beneficial for cardiovascular disease (26). A successful weight management program can help veterans to prevent weight gain, lose weight, decrease risk of long-term metabolic complications, improve quality of life and reduce the costs of medical care. These factors justify the additional expense of providing ongoing support after achievement of desired weight loss in the MOVE! program.

The prevalence of obesity among older adults has increased during the past 20 years and will affect both medical and social services. Along with an increased risk of cardiovascular disease, diabetes, and several cancers, obesity is associated with increased risk of physical and cognitive disability (27). However, little attention has been given to the issue of weight management among community-dwelling older adults. Intentional weight loss in obese older adults has not been widely advocated by health care providers because of the uncertainty of whether the benefits outweigh the risks and the potential for sarcopenia and loss of muscle mass, which can result in frailty (28). Limited data for older adults show that intentional weight loss is effective in improving physical function, quality of life, and the medical complications associated with obesity in older people (29). Therefore, weight-loss therapy that minimizes muscle and bone loss is recommended for older people who are obese and who have functional impairments or medical complications that can be ameliorated through weight loss. The veterans we studied had a mean age of 60, and the oldest participant was 92. 

Our study has limitations. We evaluated the effectiveness of MOVE! as a large-scale, hospital-based program targeting overweight and obese veterans. Although the results might underestimate intervention effects, they provide a more realistic estimate of change given the actual clinical limitations affecting both veterans and treatment providers. Furthermore, the findings from this study support the implementation of a prevention-oriented health program that has been called for by VHA policy and clinical practice guidelines. The effects of the program should be further addressed by examining the implications of weight maintenance and weight reduction on health outcomes (including medication use and number of newly diagnosed cases of diabetes or cardiovascular disease) and health-care costs. Veterans receive care, including MOVE!, without cost, but they do not receive compensation for their participation in the program as participants in randomized control trials do. Future studies should examine the effect of monetary rewards on program participation, attrition, and weight loss maintenance.

A behavioral, multidisciplinary group weight-management program for veterans implemented at VAGLAHS was effective in achieving and maintaining weight loss over a 3-year period. Findings from this study support the need for a lifestyle modification program such as MOVE! in primary care settings to assist overweight and obese veterans in managing their weight over the long term and should include a maintenance program with monthly visits.
